# Host immune responses during *Taenia solium* Neurocysticercosis infection and treatment

**DOI:** 10.1371/journal.pntd.0008005

**Published:** 2020-04-16

**Authors:** Ulrich Fabien Prodjinotho, Jakobo Lema, Matthew Lacorcia, Veronika Schmidt, Nermina Vejzagic, Chummy Sikasunge, Bernard Ngowi, Andrea Sylvia Winkler, Clarissa Prazeres da Costa

**Affiliations:** 1 Institute for Medical Microbiology, Immunology and Hygiene, Technical University of Munich (TUM), Munich, Germany; 2 Muhimbili Medical Research Centre, National Institute for Medical Research (NIMR), Dar es Salaam, Tanzania; 3 Department of Neurology, Klinikum rechts der Isar, Technical University Munich (TUM), Munich, Germany; 4 Centre for Global Health, Institute of Health and Society, University of Oslo, Oslo, Norway; 5 School of Veterinary Medicine, Department of Paraclinicals, University of Zambia, Lusaka, Zambia; 6 College of Health and Allied Sciences, University of Dar es Salaam, Dar es Salaam, Tanzania; 7 Centre for Global Health, Technical University of Munich (TUM), Munich, Germany; Hitit University, Faculty of Medicine, TURKEY

## Abstract

*Taenia solium* cysticercosis and taeniasis (TSCT), caused by the tapeworm *T*. *solium*, is a foodborne and zoonotic disease classified since 2010 by WHO as a neglected tropical isease. It causes considerable impact on health and economy and is one of the leading causes of acquired epilepsy in most endemic countries of Latin America, Sub-Saharan Africa, and Asia. There is some evidence that the prevalence of TSCT in high-income countries has recently increased, mainly due to immigration from endemic areas. In regions endemic for TSCT, human cysticercosis can manifest clinically as neurocysticercosis (NCC), resulting in epileptic seizures and severe progressive headaches, amongst other neurological signs and/or symptoms. The development of these symptoms results from a complex interplay between anatomical cyst localization, environmental factors, parasite’s infective potential, host genetics, and, especially, host immune responses. Treatment of individuals with active NCC (presence of viable cerebral cysts) with anthelmintic drugs together with steroids is usually effective and, in the majority, reduces the number and/or size of cerebral lesions as well as the neurological symptoms. However, in some cases, treatment may profoundly enhance anthelmintic inflammatory responses with ensuing symptoms, which, otherwise, would have remained silent as long as the cysts are viable. This intriguing silencing process is not yet fully understood but may involve active modulation of host responses by cyst-derived immunomodulatory components released directly into the surrounding brain tissue or by the induction of regulatory networks including regulatory T cells (Treg) or regulatory B cells (Breg). These processes might be disturbed once the cysts undergo treatment-induced apoptosis and necrosis or in a coinfection setting such as HIV. Herein, we review the current literature regarding the immunology and pathogenesis of NCC with a highlight on the mobilization of immune cells during human NCC and their interaction with viable and degenerating cysticerci. Moreover, the immunological parameters associated with NCC in people living with HIV/AIDS and treatments are discussed. Eventually, we propose open questions to understand the role of the immune system and its impact in this intriguing host–parasite crosstalk.

## Introduction

**Fig 1:**
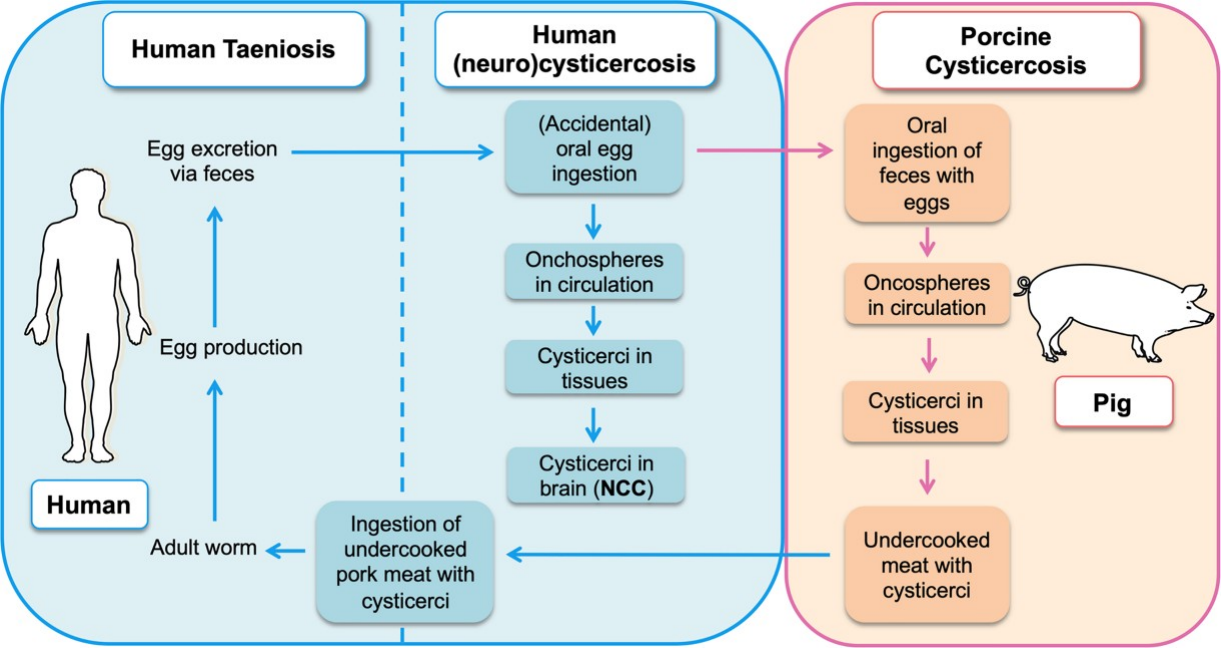
Schematic diagram of *T*. *solium* infection showing the different steps of human and porcine cysticercosis including human NCC. NCC, neurocysticercosis.

*Taenia solium* cysticercosis and taeniasis (TSCT), a neglected parasitic and zoonotic disease, is caused by the pork tapeworm *T*. *solium*. The life cycle of the parasite is complex and involves various developmental stages (eggs, oncospheres, cysticerci, and adult tapeworms). The adult stage causes *T*. *solium* taeniasis in humans, and the larval stage (cysticerci) causes porcine and human cysticercosis, which emphasizes the need for the one-health approach for the management of the disease. In the life cycle of the parasite, humans (definitive host) become infected when they consume undercooked pork meat containing cysticerci (cysts). The cysts evaginate in the upper small intestine and develop into adult tapeworms. Eggs from the adult worms, shed by a human, are ingested by a pig (intermediate host) and develop to cysticerci. When accidentally ingested by humans, eggs encyst in many tissues, particularly in the brain, and cause neurocysticercosis (NCC) (for full life cycle description, see Fig 1).

NCC is considered the most common parasitic helminth infection of the human nervous system and the most frequent preventable cause of epilepsy in endemic regions in America, Asia, and Africa, where populations are affected with symptomatic and asymptomatic infections. Although the disease is detrimental to both human and animal health, it is a preventable and potentially eradicable disease [1, 2]. The most direct approach to prevent the infection involves many conventional means, ranging from the basic improvement in sanitation and hygiene for both humans and livestock to mass drug administration and pig vaccination. Another important gap that needs to be closed is the education of affected communities to improve management of the disease.

The difficulty to eradicate NCC infection in endemic countries may also lie in the interesting fact that tapeworm carriers as key players of the transmission themselves often remain neurologically asymptomatic when affected by NCC. The reason(s) for this might also be linked to parasite-induced anti-inflammatory immunity in the host, as discussed in the section “Immune regulation during NCC.” Among other important parameters, the location (parenchyma and extraparenchyma) and type of cysts, the immune environment of the parasite as well as the interplay between the host immune cells and the excretory–secretory (ES) parasite proteins or bystander viral infections, such as HIV, are possibly important causes of the exacerbation in the infection and the different clinical presentations observed in endemic regions [3, 4]. Parenchymal and extraparenchymal NCC are two distinct diseases from clinical, immunological, and pathophysiological perspectives. Parenchymal infection is the most studied, and most available information refers to it, so the chapters in this Review document more on the infection elicited by the presence of the parasite within the brain parenchyma. How the cysticercus establishes itself in the brain, modulates the host immune responses, and survives for a lengthy period of time and how neurological symptoms eventually develop after its recognition by the host immune cells and/or treatment are scarcely known. There is a need to explore the disease interaction with the host immune system and the subsequent responses in order to, for example, identify better treatment and diagnostic tools and improve management of affected communities.

In this Review, we summarize the current literature on the immunological aspects of host–parasite interaction involved in the modulation and development of pathologies during NCC and treatment and its interaction with HIV.

## Methodology

We conducted a literature research using many electronic databases (including PubMed and Google Scholar) with the following terms in one word or in combination: *T*. *solium*, cestodes, HIV, *T*. *solium* cysticercosis and neurocysticercosis, epidemiology, global geographical distribution, clinical presentation, diagnostic, central nervous system (CNS), immune responses, immunology, immune regulation, immunopathogenesis, treatment, antihelminthic, antiretroviral, and impact. Relevant and suitable original and review articles in English on human, murine and porcine model studies were selected, and the key results and information were summarized for this Review. The online search was done prior to August 2019.

## Epidemiology and clinical presentation of TSCT and NCC

NCC is mostly endemic in rural areas of developing countries in Central and South America, Asia, and Africa, where, in many regions, it represents one of the most important burdens of neurological disease [5, 6]. It is now an increasing concern for nonendemic developed countries, mainly due to the migration of tapeworm carriers. Important risk factors for NCC include consumption of raw pork meat, free-roaming pigs, and poor sanitation. In endemic regions, 30% of all cases of acquired epilepsy are due to NCC [7]. Based on the range of epilepsy prevalence data available, 2.56 million to 8.30 million suffer from NCC worldwide [2]. A recent meta-analysis of sero-epidemiological studies on the presence of *T*. *solium*-circulating antigen and antibodies performed between 1989 and 2014 in TSCT and NCC endemic communities worldwide reported that the seroprevalence of cysticercosis in Sub-Saharan Africa ranges from 6% to 22% whereas in Asia it scales from 4% to 15.7% and to a lesser extent in Latin America from 2.5% to 6% [8–10]. In Sub-Saharan Africa, epileptic seizures are observed in 80% of symptomatic NCC individuals [11].

Symptoms and clinical presentations can vary greatly depending on several factors related either to the host (such as age, gender, geographical location, and genetic heterogeneity across regions) or to the parasite, such as number, size, type, and location of the cysts in the brain. The parenchyma is the most commonly affected site by the cysts in the brain followed by subarachnoid and intraventricular (extraparenchyma) spaces containing the cerebral spinal fluid (CSF), where they eventually degenerate, causing granuloma, cystic calcifications, and perifocal edema, which may lead to epileptic seizures and/or severe progressive headaches [10, 12]. The main symptom of parenchymal NCC is the occurrence of epileptic seizures [13, 14]. Extraparenchymal NCC is less studied and less frequent but more severe than parenchymal NCC [15, 16]. This type of NCC is more common in adult but rarely seen in children [17]. When cysts are lodged in subarachnoid and intraventricular spaces, they can become mass occupying lesions and may cause chronic inflammation at the base of the brain or obstruct the CSF, causing an increase in intracranial hypertension (the most commonly observed manifestation), arachnoiditis, headaches, hydrocephalus, and other neurological disorders [18, 19] that represent a life-threatening condition. Nevertheless, it is believed that most lesions can remain (at least clinically) silent throughout the whole life span of this cystic larval stage of the parasite [12, 20].

Another factor that may contribute to the diverse clinical presentations of the disease is coinfection with HIV and associated treatments, although, to date, there is no clear interactions demonstrated between NCC and HIV. In regions endemic for NCC, coinfected patients with HIV most frequently have multiple parenchymal lesions seen in 61% to 79.3% of cases [21]. In addition, anthelminthic and antiretroviral treatments (ARTs) could influence the course of the disease as well as the host responses and subsequent clinical presentations [3, 18].

Overall, the radiological presentations as well as the neurological symptoms are rather heterogeneous and result from the interactions of distinct factors related to the host and parasite that participate in the complex pathogenesis of the disease. Importantly, the host immune status seems to be a predisposing factor for symptomatic disease in NCC patients and will thus be the focus of this Review.

## Host immune responses and pathogenesis during NCC

The host response to the cyst is remarkably complex and diverse. Typically, the immune response to the parasite is of type 2 (as observed in the majority of affected individuals with asymptomatic infection associated with immune regulation) but can change during symptomatic diseases. Although NCC is usually described as a unique disease, it encompasses two distinct diseases (parenchymal and extraparenchymal NCC) with respect to the location of the cyst in the brain and the corresponding immune responses. In the following two chapters, we have summarized the current knowledge on immune responses, namely the humoral and cellular, during NCC alongside the associated pathologies and clinical presentations with a particular highlight on parenchymal NCC.

### Humoral immune responses in NCC

Numerous immunodiagnostic assays rely on the humoral immune responses raised against *T*. *solium* cysts and oncospheres. Indeed, invasive oncospheres are susceptible to antibody-mediated parasite neutralization and complement factors [22]. The role of the complement system in NCC is not yet fully understood. A recent investigation has associated the complement component C5 haplotype CAA with extraparenchymal infection [23]. Furthermore, previous investigations suggest that the complement system may be involved in the activity of antibodies generated during NCC [22, 24]. Evidence of the role of antibodies in NCC, although up to now not clearly specified, has been demonstrated by the presence of cluster of differentiation 20 (CD20) expressing B and plasma cells and the identification of Russell bodies (eosinophilic inclusions of antibody) within plasma cells close to the brain lesion of NCC [25, 26]. Several antibody classes are produced during the clinical course of NCC, with the most prominent being IgG in serum and CSF. In extraparenchymal infection, increased levels of immunoglobulin G (IgG), immunoglobulin M (IgM), and immunoglobulin E (IgE) were observed in symptomatic patients harboring cysts in subarachnoid spaces compared to asymptomatic NCC individuals [27–29]. Non-NCC subjects from high-endemic areas had higher levels of specific IgG1, IgG2, IgG4, and IgE antibodies than subjects from low-endemic areas [27]. This indicates that antibody responses relate to the intensity of exposure and that some non-NCC subjects from endemic areas are exposed to *T*. *solium* but have a strong immunity that could prevent parasite establishment. Other studies suggested the influence of the developmental stage of the parasite on the presence of antibodies, especially IgG1 and IgG4, in CSF and in serum [30, 31]. Thus, the parasite load and radiological presentations of patients contribute to the humoral responses to NCC. This is associated with the exposure and intensity of infection as well as the developmental stage of the parasite and induced cellular immune responses.

### Cellular immune responses in NCC

Cellular immune responses during NCC are diverse and distinct with regard to the localization and stages of parasite in the brain and mostly limit the progression of the disease [14, 32]. After cysticerci reach their final location in the parenchyma of the brain, there usually follows a period of several months or years until the onset of symptoms. During this period, affected individuals possibly remain asymptomatic through active evasion and suppression of host immunity by viable cysticerci (detailed in the chapter below) since the development of symptoms has been shown to strongly depend on the degree and intensity of the host response [28]. Indeed, the cytokines produced by cells within the brain in response to chronic NCC strongly regulate local tissue damage and development of the pathology. After months to years in the brain tissue, the cysticerci start to degenerate, either naturally (the precise cause is not known) or due to anthelmintic treatment, and lose their ability to regulate the host response. Thus, an inflammatory immune response characterized by an increase in interleukin 1 beta (IL1ß), tumor necrosis factor alpha (TNFα), and interferon gamma (IFNγ) production is instigated [25, 33]. These cytokines can trigger astrocyte proliferation and gliosis and have been shown for example to alter the physiology of the hippocampus and, thus, may influence depressive symptoms and pathogenesis such as hippocampal sclerosis, although no clear correlation is established to date [33–35]. This inflammatory response is followed by an intense activation of endothelial and associated cells such as astrocytes located in the blood–brain barrier (BBB) [36]. These cells are interlinked by tight junctions formed by transmembrane and junctional adhesion molecules. The uncontrolled activation of endothelial and associated cells of the BBB disrupts the junction and formation of *trans*-endothelial cell channels and thereby the integrity of the BBB [37]. Then, activated endothelial cells and affected circulating leukocytes up-regulate many integrins and adhesion molecules such as P-selectin, the vascular cell adhesion molecule 1 (VCAM1), and the intercellular adhesion molecule 1 (ICAM1) as well as the chemoattractants C-X-C ligand (CXCL)2, leukotriene B4 and complement component C5a and the chemokines C-C ligand (CCL)5, CCL2, and CCL20 (including their receptors CCR2 and CCR6, which play a major role in neutrophil and monocyte mobilization) [38–40]. These processes eventually facilitate the infiltration and migration of leukocytes across and through the BBB into the subarachnoid space containing CSF via the choroid plexus and into the brain parenchyma via the perivascular space. Thereby, immune cells eventually reach the cysts and get into contact with the cysts or cystic material.

Histological and immunohistochemical analysis of patient’s brain tissue revealed that degenerating (colloidal and granular-nodular) and calcified cysts are surrounded by a mature granuloma and the architecture of the granuloma is composed of, from the inner region close to the parasite to the outer region, multinucleated giant cells surrounded by a dense fibrous layer rich in collagen, a leukocyte-rich region with infiltrating CD68 expressing macrophages found close to the fibrous layer, T and B lymphocytes (namely CD3^+^, CD4^+^, and abundant CD8^+^ and CD20^+^ cells), plasma cells, neutrophils, mast cells, eosinophils, and brain microglia [25, 26, 41]. However, the role of brain cells (e.g., microglia) during NCC and the dynamic of leukocyte infiltration and subsequent granuloma formation are not really known. This important leukocyte infiltration in the brain may lead to an increase in intracranial pressure, headaches, and encephalitis observed in NCC patients [18, 42]. Indeed, once the cysts are surrounded and infiltrated by these inflammatory cells, the cysts pass through a series of involuting stages associated with inflammation in the brain. Consequently, the integrity of the cyst is altered, its cavity invaded by inflammatory cells (colloid stage), and potentially immunogenic cyst products (e.g., cyst vesicular fluid, tegument antigens, and excretory-secretory (ES) products) are released. Alongside this inflammation, the secretion of Substance P (SP), a neuropeptide of the tachykinin family produced by neurons, endothelial cells, and lymphocytes, is observed [43]. The secretion of SP is tightly associated with the expression of inflammatory type 1 cytokines such as IFNγ, IL-2, IL-12, IL-18, and TNFα found in the CSF and in lesions surrounding dying cysts and TNFα, IL-1ß, and IL-6 in periphery [25, 26, 43, 44]. Recent findings indicate that SP is a critical mediator of seizures in NCC and an inducer of inflammatory T helper cell 1 (Th1) cytokines and granuloma formation [43–45]. As the host response progresses, fibrosis encompasses the cyst, and it contracts, becomes nodular, and the scolex granulated (granular-nodular stage). This stage is associated with a mixed Th1 and Th2 immune response characterized by IFNγ, IL-18, IL-4, IL-10, and IL-13 production associated with an increased amount of the fibrinogenic cytokine transforming growth factor beta (TGF-ß) [26, 45]. IL-4 and IL-13 may play an essential role in the formation of mature granuloma and TGF-ß in fibrosis development observed at this stage. Then, there is the formation of collagenous structures, associated with TNFα, IL-1ß, IL-4, IL-6, and IL-10 [25, 46], and the parasite is replaced by progressive fibrosis, which may calcify (calcified stage). Calcified lesions have an important role in the occurrence of seizure episodes, as a positive correlation has been observed between cerebral calcifications and seizure activity in symptomatic patients [12, 47]. However, asymptomatic patients with calcified lesions exhibited a dominant Th2 response with high levels of IL-4, IL-5, and IL-13 in antigen-specific in vitro studies [27] and significantly increased serum levels of IgG4, IL-4, and IL-10 [42]. In some patients, this stage can lead to occlusion of cerebral arteries and infarction [48]. Thus, classically, during the course of cyst involution, the initial, predominantly Th1 inflammatory response, with disruption of the BBB, evolves into a chronic Th1 and Th2 phenotype associated with a mature granuloma formation, fibrosis, and angiogenesis. Since the BBB is disrupted, released cyst antigens may reach the periphery, where a systemic immune response is induced.

**Fig 2:**
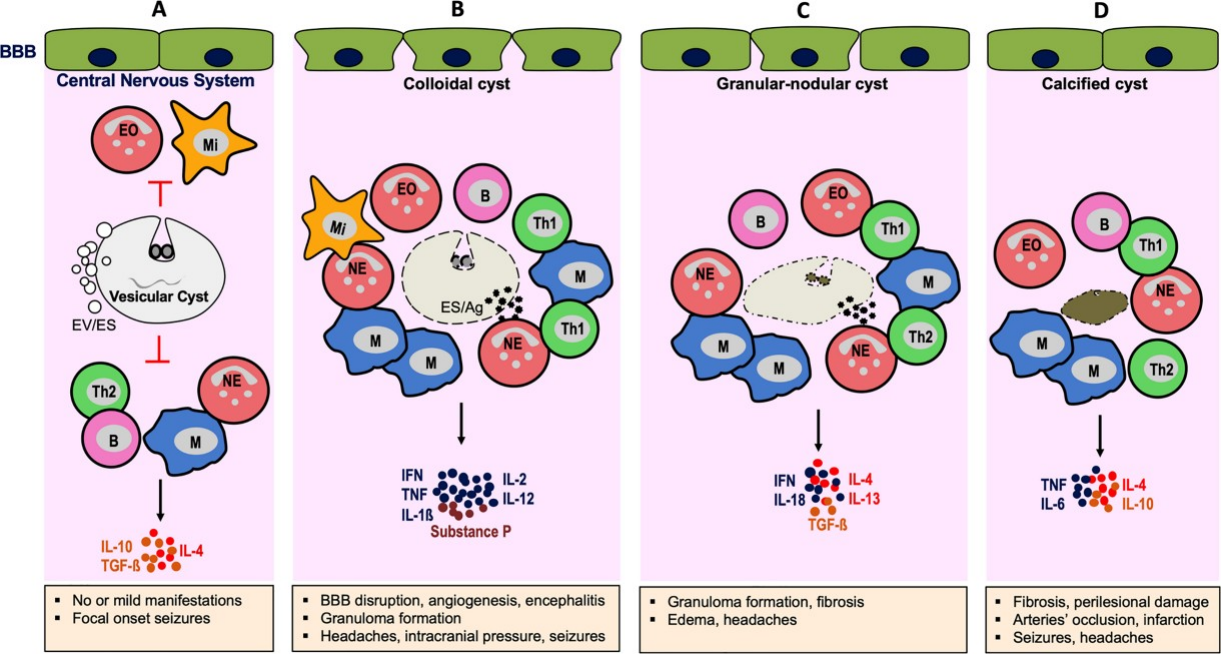
Cyst involution in brain parenchyma and associated immunopathogenesis. The presence of viable vesicular cysts (A) is usually asymptomatic and associated with suppression of the host immune responses through induction of anti-inflammatory cytokines. Upon successful attack of the host immune system, the cyst passes through a series of involuting stages (B–D). The BBB is disrupted, and granuloma, mainly composed of macrophages, granulocytes, and lymphocytes, encompasses the cyst that contracts (colloidal stage) (B) and becomes nodular and the scolex granulated (granular-nodular stage) (C); this is followed by the formation of collagenous and fibrotic structures and calcification of the cyst (calcified stage) (D). Each involuting stage of the cyst releases material (ES and Ag) and elicits an inflammatory reaction and release of mediators in the brain that lead to the development of pathology and symptoms (pale orange box). Ag, cyst antigen; B, lymphocyte B; BBB, blood–brain barrier; EO, eosinophil; ES, excretory–secretory; EV, extracellular vesicle; IFN, interferon; IL, interleukin; M, macrophage; Mi, microglia/dendritic cell; NE, neutrophil; TGF-ß, transforming growth factor beta; Th, T helper cell; TNF, tumor necrosis factor.

In addition, extraparenchymal NCC is associated with an increase in cell numbers and parasite antigen levels and strong antibody reactions [13, 49]. Thus, high numbers of lymphocyte CD8^+^ T cells and neutrophils have been found to be elevated surrounding extraparenchymal cysts [25, 26]. Furthermore, increased CSF levels of IL-1ß, IL-5, IL-6, IL-10, IL-12, and IgG subclasses were detected locally in patients with CSF inflammation [50, 51]. This was associated with increased reactive oxygen species, glial fibrillary acidic protein, CSF lipoperoxidation, cell counts (CSF pleocytosis) [52, 53], and, importantly, arachnoiditis formation [13, 14, 50]. This can disturb the normal flow of the CSF, leading to intracranial pressure, cerebral infarction, and perilesional edema associated with seizure episodes. Further mechanisms are being explored to understand the complex neuropathology and immunopathology as well as the heterogeneity of NCC and the cellular and humoral immune responses involved. The currently known mechanistic pathways during NCC and commonly reported manifestations are presented in Fig 2.

With regards to the number of cysts and related immunopathology, single (degenerating) cyst infections usually elicit a proinflammatory Th1 response associated with IFNγ, IL-12, and IL-18 production in the periphery and development of symptoms [54, 55]. In contrast, patients with multiple (viable and degenerating) cysts exhibit an anti-inflammatory Th2 immune response characterized by prominent expression of IL-4, IL-5, IL-6, IL-10, and IL-13 cytokines and lower levels of effector memory CD4^+^ T cells [54, 55]. Thus, a shift from Th1 to Th2 cytokine response may even favor the establishment of multicyst infection and can be the result of active regulation of Th1 immune response by the parasite alongside Th2-mediated repair mechanisms such as fibrosis. (See Table 1 for a summary of the characteristics and key effectors of cellular and humoral responses during NCC.)

**Table 1:** Key cellular and humoral effectors associated with parenchymal and extraparenchymal NCC.

	Parenchymal NCC	Extraparenchymal NCC	References
**Humoral response**	IgG1, IgG2, IgG4, IgE (Serum/plasma)	IgG, IgM, IgE (CSF) Complement (C5 haplotype CAA)	23, 27, 29, 30, 54
**Cellular response**	Microglia/Macrophages (CNS) Mast cells (CNS) Neutrophils/Eosinophils (CNS) CD20^+^B/Plasma cells (CNS) CD8^+^T cells (CNS) NK cells (CNS) CD4^+^CD25^high^Treg (Periphery) Dendritic cells (Periphery)	Macrophages (CNS) Mast cells (CNS) Neutrophils B/Plasma cells (CNS) CD8^+^T cells (CNS) CD4^+^CD25^+^Treg (Periphery) Dendritic cells (Periphery)	25, 26, 41, 42, 64, 65
**Cytokines**	IL-4, IL-6, IL-10, TGF-ß (VC) IL-1ß, IL-2, IL-12, IL-4, IL-6, IL-13, IL-18, TNFα, IFNγ (DC) IL-4, IL-6, IL-10, TNFα (CC) Serum/Plasma/Brain parenchyma	CSF: IL-1ß, IL-5, IL-6, IL-10, IL-12 (VC/DC/CC) Serum/Plasma: IL-4, TNFα, IFNγ, IL-10 (VC/DC/CC)	25, 26, 27, 42, 43, 50, 51, 54, 55
**Main manifestations**	Seizures, perilesional edema, headaches, focal deficits, cognitive decline	Focal deficits, arachnoiditis, occlusion of arteries, hydrocephalus, intracranial hypertension, focal deficits, cerebral infarcts	10, 14, 16, 19

CAA, complement C5 haplotype; CC, calcified cyst; CD4/8/20/25, cluster of differentiation 4/8/20/25; CNS, central nervous system; CSF, cerebral spinal fluid; DC, degenerating cyst; IFNγ; interferon gamma; IL, interleukin; NCC, neurocysticercosis; NK, natural killer; TGF-ß, transforming growth factor beta; TNFα, tumor necrosis factor alpha; Treg, regulatory T cells; VC, vesicular cyst

## Immune regulation during NCC

*T*. *solium* cysts are known to induce persistent infections that can last for decades in humans. Establishing such long-term infections requires modulating the host immune system for a long period of time and suggests the existence of a vast array of immunoregulatory mechanisms, principally observed in asymptomatic infected patients. Although immune modulation by the parasite can be beneficial to the host in term of avoiding uncontrolled inflammatory responses, it may also turn to be detrimental for the host, especially during therapies and coinfection setting, as it can affect cellular as well as humoral immune responses to bystander infections.

### Regulation of humoral immune responses

A well-known mechanism employed by cysts to enable survival in immunologically privileged sites is masking cysticercal antigens by host immunoglobulins. Thus, cysts have been shown to harbor IgG, IgM, IgA, and IgE on their tegument, probably acquired by fragment crystallizable (Fc)-receptor–mediated endocytosis [42, 56]. Another key strategy developed by the parasite to control innate immunity is to block the complement system by released antigens. For example, taeniastatin from cysts inhibits both classic and alternative complement pathways while paramyosin binds to complement first component C1q and inhibits its activity [57, 58]. The live cyst is also known to secrete cysteine proteases, metallo proteases, and serine proteases that degrade host immunoglobulins and interfere with CD4^+^ cell proliferation and cytokine production and, thus, influence antibody production by B cells [59].

### Regulation of cellular immune responses

Classically, *T*. *solium* parasites, as reported for many helminth parasites, induce a typical type 2 immune response associated with cytokines displaying immunoregulatory properties such as TGF-ß and IL-10 [27]. These immunoregulatory properties affect, on the first line, cells of the innate immunity such as dendritic cells (DCs), macrophages, and monocytes, which play a key role in the limitation of the progression of the infection.

The presence of DCs in the brain during NCC is evident, but the isolation and investigation with these cells are extremely challenging, and, thus, DCs generated from human peripheral blood or from mouse tissues such as the bone marrow are often used to explore the regulation of DC function in NCC. DCs are professional in processing and presenting antigens from helminth parasites to naïve and memory T cells. This important task for the control of the infection can only be done when DCs reach maturity characterized by the high expression of CD40, major histocompatibility complex class II (MHC-II) antigens, and the costimulatory molecules CD80 and CD86. Cysticerci ES have been found to block this process to evade host immune responses [60]. Previous investigations demonstrated that in *T*. *crassiceps* infection and in experimental *Echinococcus multilocularis* (another genera of the Taeniidae family) the maturation of peritoneal and monocyte-derived DCs is impaired, with diminished expressions of CD80, CD86, and MHC-II but increased levels of TGF-ß and IL-10 [60, 61]. Furthermore, these cells do not respond to lipopolysaccharide (LPS), and thus to Toll-like receptor 4 (TLR4) stimulation, and display impaired expression of proinflammatory cytokines TNFα and IL-12 [60]. Indeed, it has been shown that, upon recognition of *T*. *crassiceps* glycoconjugates by lectin, mannose, and Toll-like receptors on DCs and macrophages, there is an induction of the extracellular-signal-regulated kinase 1 and 2 (ERK1 and ERK2) signaling, which blocks LPS-associated phosphokinase signaling pathways [60]. In addition, a detailed immunohistochemical analysis of human brain affected with NCC localized TGF-ß production in DCs and macrophages within the granuloma surrounding cysticerci [26].

Immunomodulation of macrophages through the induction of alternatively activated macrophages (AAMs) that lead to the production of immunosuppressive cytokines such as TGF-ß and IL-10 and activation of the arginase-1 (Arg-1), chitinase-like protein YM1, and resistin-like protein Fizz1 pathways has also been suggested as an immune-protective mechanism employed by the parasite [62]. Thus, high levels of the immunoregulatory cytokine TGF-ß are detected in regions rich in macrophages in the brain of NCC patients [26]. Furthermore, macrophages found during infection of mice with *T*. *crassiceps* are characterized by high expression of IL-4, programmed death ligand 1 (PD-L1), and programmed death ligand 2 (PD-L2), and inducible nitric oxide synthase (iNOS) but low levels of IL-12 [62, 63], rendering those macrophages as potent suppressors of lymphocyte proliferation, adhesion molecules, and chemokine expression. In addition, to induce a decrease in lymphocyte proliferation, cysticerci were also shown to be prominent inducers of apoptosis of eosinophils and inhibitors of microglia and granulocyte aggregation and chemotaxis [58, 62].

Another important mechanism of the modulation of host immunity by viable cysts is the induction of regulatory T cell (Treg) activity, both at the central and peripheral level. It is currently hypothesized that after disruption of BBB, cysts or rather cyst-related antigens are released into the peripheral system and thus lead to an increase of Treg activity, likely as a mechanism to limit the inflammatory response in CNS [62, 64]. In vitro studies have demonstrated that cysticerci promote immature or semimature DCs, which up-regulate the expression of IL-10, signaling lymphocytic activation molecule 1 (SLAMF1), B7 homolog 1 (B7-H1), and CD205, to convert naïve CD4^+^ cells into CD25^+^Foxp3^+^ Tregs. These express high levels of IL-10, cytotoxic T-lymphocyte antigen 4 (CTLA-4), programmed cell death protein-1 (PD-1), and glucocorticoid-induced tumor necrosis factor receptor (GITR) and thus favor the suppression of neuro-inflammation and establishment of multiple cysticerci [62, 64, 65]. Until now, however, it is unknown which parasitic molecules are responsible for these effects, and, furthermore, evidence is still lacking on whether the same mechanisms apply to NCC in humans.

**Fig 3:**
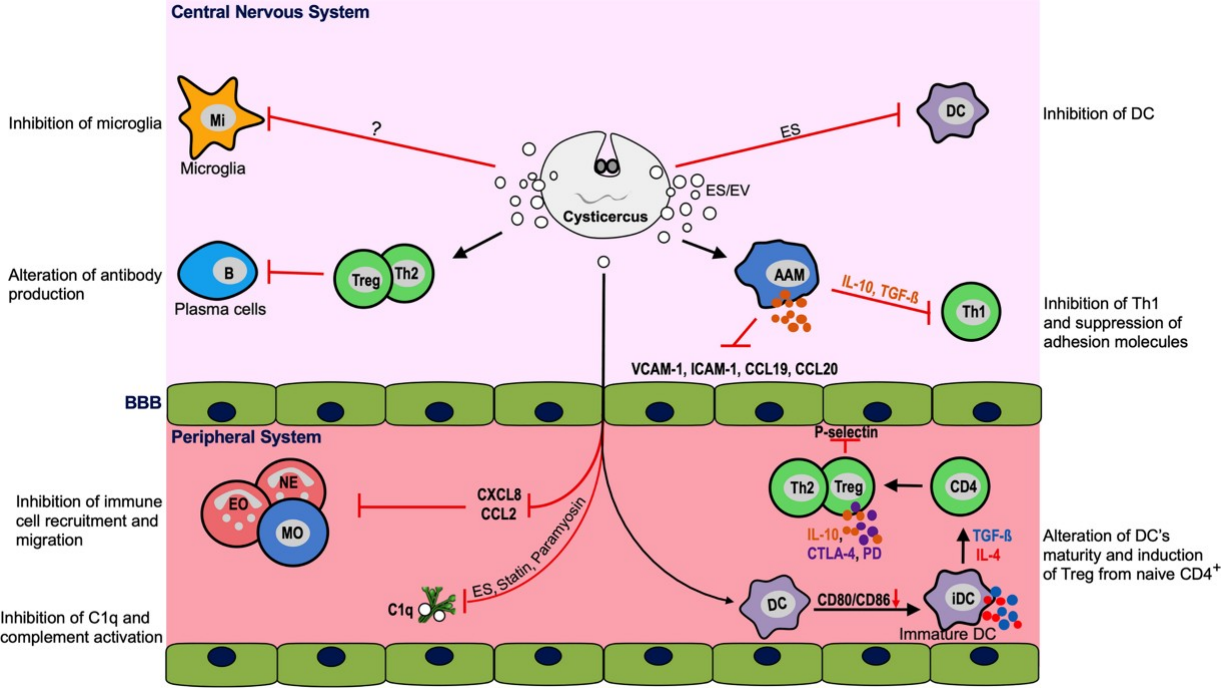
Host immune regulation during parenchymal NCC. Viable cyst releases ES products and EVs that interact with resident brain cells (microglia and DCs) and induce AAMs, which suppress adhesion molecules and local Th1 response via TGF-ß and IL-10. Excreted products reach peripheral system where they drive the expansion of suppressive Tregs from CD4^+^ cells through the alteration of the maturation of DCs. Furthermore, cyst products inhibit complement C1q activity and prevent the infiltration and migration of neutrophils, eosinophils and MO from the peripheral system into the brain via production of immunomodulatory cytokines and blocking of chemokines and adhesion molecules. AAM, alternatively activated macrophages; B, ; BBB, blood–brain barrier; CCL, chemokine C-C ligand; CD4, cluster of differentiation 4; C1q, complement first component; CTLA-4, cytotoxic T-lymphocyte associated protein 4; CXCL, chemokine C-X-C ligand; DC, dendritic cell; EO, eosinophil; ES, excretory–secretory; EV, extracellular vesicle; ICAM-1, intercellular adhesion molecule 1; iDC, immature DC; IL, interleukin; Mi, microglia; MO, monocytes; NCC, neurocysticercosis; TGF-ß, transforming growth factor beta; Th, T helper; Treg, regulatory T cell; VCAM-1, vascular cell adhesion protein 1.

The main pathways currently proposed to be involved in the regulation of host immune responses by cysticerci-related products are reviewed in Fig 3. In addition to these regulatory mechanisms associated with the parasite, anthelmintic treatment during the infection has been shown to profoundly affect the host immune response.

## Anthelmintic treatment, HIV, and impacts on the immune response during NCC

The two main anthelminthic drugs used in the management of NCC are albendazole (ABZ) and praziquantel (PZQ), which are given along with antiepileptic drugs such as phenobarbitone, phenytoin, or carbamazepine, as well as anti-inflammatory drugs and analgesics depending on the symptoms of the patient. However, how these anthelminthic drugs impact on the host immune response is not clearly understood. Classically and as mentioned above, most asymptomatic viable cysts induce type 2 immune responses with prominent levels of IgE and IgG1. During anthelmintic treatment, the immune response switches to a type 1 profile, following cyst degeneration, with a prominent inflammatory response directed against the parasite [18]. This response has been suggested to be partly mediated by the myeloid differentiation primary response 88 (MyD88) pathway, which plays a role in cellular infiltration and BBB permeability and is a key regulator of cytokines such as TNFα, IL-1ß, and IL-6 produced by monocyte-derived cells [66].

To reduce the intensity of the type 1-mediated inflammation, which may severely damage brain tissues, combination therapy is recommended (especially for patients with solitary cysticercus granuloma) but might need to be implemented case by case [9, 19, 67]. Thus, ABZ and PZQ are generally used in combination with anti-inflammatory drugs such as corticosteroids. This combination generates a mixed type 1 and type 2 immune response in the porcine model of NCC, characterized by low levels of TNFα, IFNγ, and IL-1ß and high levels of IL-4, IL-6, and IL-10, which may dampen the inflammation [18, 68]. In addition, in a human study, responders to combination therapy showed higher CSF IL-17AF levels 60 days after treatment [69]. Consequently, combination therapy often prevents seizure recurrence and promotes lesion resolution but reduces the clearance rate of cysts as demonstrated in patients with multicyst infections [18, 42]. So, treatment of patients with corticosteroids can have detrimental effects on the host immune responses. These include the inhibition of lymphocyte binding to endothelial cells, increased apoptosis of T and B cells in the thymus and the periphery, and inhibition of TLR pathways in macrophages and DCs via direct suppression of p38/MAPK phosphorylation, which results in down-regulation of DC migration and maturation [70, 71]. These alterations are associated with the inhibition of the activity of several inflammatory cytokines including IL-1β, IL-2, IL-6, IL-12, iNOS, IFNγ, and TNFα and the reduction of antibody responses [70, 71]. These treatment-associated alterations of host immune responses could potentially elicit bystander effects on other chronic infections such as HIV infections in coinfected individuals.

Like *T*. *solium* infection, HIV infection is also a leading cause of chronic immune activation in the CNS [72]. The prevalence of HIV in regions endemic for NCC is high [73]. Despite the presence of symptoms in NCC and HIV patients, little is known about the influence of HIV infection on the frequency, clinical course, and pathogenesis of NCC. A few number of NCC patients coinfected with HIV with very low CD4^+^ cell counts and high viral loads commonly do not develop cerebral symptoms, but, after initiation of ART, these patients develop a profound pathologic inflammatory reaction characterized by a sudden increase in activated immune memory CD4^+^CD45RO^+^ cells with a significant CD8^+^ cytotoxic T cell response associated with CSF inflammation [3, 74]. Nevertheless, very early initiation of ART in HIV infection only has been shown to normalize CSF immune activation markers such as neopterin, CXCL10, CCL2, and IL-6 and related inflammation [75]. Although some studies suggested that patients at very early stage of HIV infection with higher CD4^+^ T-lymphocyte counts are more likely to develop symptomatic NCC and that NCC may dampen both protective and immunopathological responses to HIV and can have a role in HIV acquisition and dissemination in infected patients [74, 76], there is no clear evidence that the immunosuppression induced by HIV facilitates NCC and that NCC infection can ease or exacerbate HIV [21]. In line with this, a cross-sectional study in Tanzania demonstrated that, among HIV+ patients, cysticercosis infection was not associated with CD4^+^ cell counts, ART duration, or HIV stage [73]. Thus, further investigations are needed to characterize the pathogenesis and therapeutic response of NCC in the setting of HIV infection and during treatment and identify more efficacious and safe anthelmintic agents and better treatment combinations and regimens to minimize the impact on the host immune system and development of pathology.

## Conclusion and outstanding questions

The immunology of *T*. *solium* infection is diverse and complex and depends on the combination of several variables. These include the number, localization, size, and developmental stage of the parasite as well as host-related factors, which can affect the course and severity of disease manifestations. Host-related factors determine susceptibility and the degree as well as the type of the initial and developing immune responses. The interaction of these factors generates a complex host–parasite interplay, which may drive pathology, as in symptomatic patients, or down-regulate inflammation, as in most asymptomatic individuals. This interplay could be further disturbed during HIV and NCC coinfection, in which the CD4^+^ T cells, as well as innate immune responses, could be compromised. Since many of the underlying mechanisms that influence the nature of the host immune responses still remain elusive, we propose to fill the following gaps in our knowledge of this intricate host–parasite crosstalk: (A) What role do the innate immune cells of the brain (the primary site of symptomatic human NCC), e.g., microglia, play in orchestrating local and systemic immune responses? (B) How do the parasites remain viable in the CNS without recognition from host immune cells in asymptomatic patients? (C) Do the secreted products of cysts change with aging and what specific mechanisms initiate the immune attack that triggers cyst degeneration and inflammation? (D) How do host innate and adaptive immune cells react to different developmental stages of cyst and related antigenic preparations (e.g., vesicular fluid versus tegument)? (E) Besides Tregs, what role do other regulatory cells like Bregs or AAMs play? (F) Do different strains of *T*. *solium* cysts cause different immune responses? (G) Do the parasite ES products harbor molecules homologous to host inhibitory molecules, and, if so, can comparative proteomic studies expand our understanding of the underlying mechanisms that drive inflammation or down-regulation in NCC?

The answers to these basic questions will be paramount to design novel vaccines, new diagnostic tests, and treatments, which side by side will help to control and eventually eliminate TSCT and NCC.

**Key learning points**

1. NCC, caused by the larval cyst of the pork tapeworm *T*. *solium*, is a clinically and radiologically pleomorphic disease of the human brain.
2. NCC is one of the leading causes of acquired epilepsy in endemic countries.
3. Host immune responses to viable and degenerating cysts are heterogeneous and possibly due to differential immune suppression or activation, respectively.
4. Treatment of NCC, especially in coinfection with HIV, may have detrimental effects due to overwhelming immune responses and, thus, needs to be considered.

**Top five papers**

1. White AC, Jr., Coyle CM, Rajshekhar V, Singh G, Hauser WA, Mohanty A, et al. Diagnosis and Treatment of Neurocysticercosis: 2017 Clinical Practice Guidelines by the Infectious Diseases Society of America (IDSA) and the American Society of Tropical Medicine and Hygiene (ASTMH). Am J Trop Med Hyg. 2018;98(4):945–66. doi: 10.4269/ajtmh.18-88751. PubMed PMID: 29644966; PubMed Central PMCID: PMCPMC5928844
2. Garcia HH, Nash TE, Del Brutto OH. Clinical symptoms, diagnosis, and treatment of neurocysticercosis. Lancet Neurol. 2014;13(12):1202–15. doi: 10.1016/S1474-4422(14)70094-8. PubMed PMID: 25453460; PubMed Central PMCID: PMCPMC6108081
3. Fleury A, Trejo A, Cisneros H, Garcia-Navarrete R, Villalobos N, Hernandez M, et al. Taenia solium: Development of an Experimental Model of Porcine Neurocysticercosis. PLoS Negl Trop Dis. 2015;9(8):e0003980. doi: 10.1371/journal.pntd.0003980. PubMed PMID: 26252878; PubMed Central PMCID: PMCPMC4529300
4. Tuero I, Palma S, Cabeza F, Saleemi S, Rodriguez S, Gonzales I, et al. A Comparative Study of Peripheral Immune Responses to Taenia solium in Individuals with Parenchymal and Subarachnoid Neurocysticercosis. PLoS Negl Trop Dis. 2015;9(10):e0004143. doi: 10.1371/journal.pntd.0004143. PubMed PMID: 26506532; PubMed Central PMCID: PMCPMC4624727.
5. Schmidt V, Kositz C, Herbinger KH, Carabin H, Ngowi B, Naman E, et al. Association between Taenia solium infection and HIV/AIDS in northern Tanzania: a matched cross sectional-study. Infect Dis Poverty. 2016;5(1):111. doi: 10.1186/s40249-016-0209-7. PubMed PMID: 27903304; PubMed Central PMCID: PMCPMC5131417.
